# Hepatoprotective Effects of *Berberis vulgaris* L. Extract/β Cyclodextrin on Carbon Tetrachloride–Induced Acute Toxicity in Mice

**DOI:** 10.3390/ijms13079014

**Published:** 2012-07-19

**Authors:** Anca Hermenean, Cristina Popescu, Aurel Ardelean, Miruna Stan, Nicoleta Hadaruga, Ciprian-Valentin Mihali, Marieta Costache, Anca Dinischiotu

**Affiliations:** 1 Department of Histology, Faculty of Medicine, Pharmacy and Dentistry, Vasile Goldis Western University of Arad, 1 Feleacului, Arad 310396, Romania; 2 Department of Experimental and Applied Biology, Institute of Life Sciences, Vasile Goldis Western University of Arad, 86 Rebreanu, Arad 310414, Romania; 3 Department of Biochemistry and Molecular Biology, University of Bucharest, 91-95 Splaiul Independentei, Bucharest 050095, Romania; 4 Food Quality Department, Faculty of Food Processing Technology, Banat’s University of Agricultural Sciences and Veterinary Medicine, 119 Calea Aradului, Timisoara 300645, Romania

**Keywords:** *Berberis vulgaris* extract, β-cyclodextrin, hepatoprotection, oxidative stress

## Abstract

The present study investigated the capacity of formulated *Berberis vulgaris* extract/β-cyclodextrin to protect liver against CCl_4_-induced hepatotoxicity in mice. Formulated and non-formulated extracts were given orally (50 mg/kg/day) to mice for 7 days and were then intra-peritoneally injected with 1.0 mL/kg CCl_4_ on the 8th day. After 24 h of CCl_4_ administration, an increase in the levels of apartate-amino-transferase (AST), alanine-amino-transferase (ALT) and malondialdehyde (MDA) was found and a significant decrease in superoxide-dismutase (SOD), catalase (CAT), glutathione (GSH) and glutathione-peroxidase (GPx) levels could be detected. This was accompanied by extended centrilobular necrosis, steatosis, fibrosis and an altered ultrastructure of hepatocytes. Pre-treatment with formulated or non-formulated extract suppressed the increase in ALT, AST and MDA levels and restored the level of antioxidant enzymes at normal values. Histopathological and electron-microscopic examination showed milder liver damage in both pre-treated groups and the protective effect was more pronounced after the formulated extract was administered. Internucleosomal DNA fragmentation induced by CCl_4_ was reduced in the group which received non-formulated extract and absent in the group which received formulated extract. Taken together, our results suggest that *Berberis vulgaris*/β-cyclodextrin treatment prevents hepatic injury induced by CCl_4_ and can be considered for further nutraceutical studies.

## 1. Introduction

The liver is prone to xenobiotic-induced injury because of its central role in xenobiotic metabolism and its portal location within circulation [1].

Several xenobiotics, such as: arsenic, cadmium, chloroform, carbon tetrachloride, pesticides, dioxins, nitrosamines [2] become hepatotoxic through metabolisation and bioactivation processes. The first phase reactions (oxidation, reduction or hydrolysis, *etc.*), followed by conjugation ones, play an important role in converting xenobiotics into a form suitable for elimination in order to prevent xenobiotic-induced liver injury [3,4]. But these metabolic processes require multiple biochemical transformations, and in some cases, the intermediates mediate toxic responses. Therefore, the liver is potentially susceptible to injury during the act of performing its function [5].

Carbon tetrachloride (CCl_4_) is the best characterized model of xenobiotic-induced hepatotoxicity and commonly used for the screening of hepatoprotective effects of drugs and natural products. Liver damage induced by carbon tetrachloride (CCl_4_) is based on metabolisation to trichloromethyl radicals (CCl_3_· and/or CCl_3_OO·) under cytocrome P450 action [6,4].

Many herbs are used by traditional medicine to prevent and treat liver diseases. Several hundred plants have been examined for use in a wide variety of liver disorders, but just a part have been well investigated, such as: *Silybum marianum* (milk thistle), *Picrorhiza kurroa* (kutkin), *Curcuma longa* (turmeric), *Camellia sinensis* (green tea), *Chelidonium majus* (greater celandine), *Glycyrrhiza glabra* (licorice), *Allium sativa* (garlic) [7–9].

Berberry (*Berberis vulgaris* L., family Berberidaceae) is a herb growing in Europe and Asia, which has been less investigated from a therapeutic point of view. The root, bark, leaf and fruit have been used in traditional medicine to treat diarrhea, colitis, gastroenteritis, and hepatic disorders. Several alkaloid constituents with an isoquinolinic nucleus, such as berberine, berbamine and palmatine were isolated [10]. Other compounds like terpenoids lupeol, oleanolic acid, stigmasterol and stigmasterol glucoside [11] as well as polyphenols [12] were also identified. Nevertheless, berberine is the most important alkaloid that is generally claimed to be responsible for their beneficial effects [12]. There are multiple pharmacological effects of berberine, such as antimicrobial [13,14], anti-tumor [15–17], and anti-inflammatory effects [18–20]. It also has effects on the gastro-intestinal [11,21–23], cardiovascular [24–26] and nervous [27] systems.

Several studies revealed that berberine exerts preventive and curative effects on liver against experimental injuries. Feng *et al* [28] reported a significant decrease of hepatic marker enzymes in CCl_4_ treated rats after oral administration of berberine at 80, 120, 160 mg/kg daily doses compared to control animals, whereas a lower dose (4 mg/kg) was not effective [29]. On the other hand, intraperitoneal administration of berberine in rats in a dose of 0.5–5 mg/kg counteracted the damaging effect of tert-butyl hydroperoxide by reducing the generated oxidative stress [30]. A few studies concerning the oral treatment of rats with extract of *Berberis vulgaris* root have been reported [31]. The dose that effectively protected the liver was 900 mg/kg, 30 times higher than average dose (THD) used in some traditional systems of medicine.

There is no report in the literature on the protective effect of formulated *Berberis vulgaris* L. extract/β-cyclodextrin against CCl4-induced liver injury. Therefore, the present study was carried out to evaluate the preventive effect of *Berberis vulgaris* L. extract against CCl_4_-induced acute hepatotoxicity in mouse liver as well as the effects of β-cyclodextrin complexation, in hepatoprotective therapeutical formulations.

## 2. Results and Discussion

### 2.1. The Complex of *Berberis vulgaris* Extract and β Cyclodextrin

**Figure 1:**
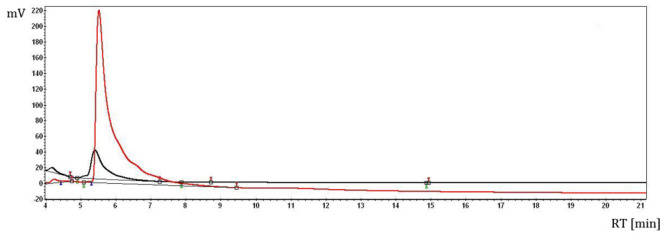
HPLC analysis of the re-extracted (ethanolic solution) *Berberis vulgaris* L. sample from the β-cyclodextrin complex (lower chromatogram—black) in comparison with the berberine standard solution (upper chromatogram—red).

The presence of the berberine (the main compound from *Berberis vulgaris* L. bark) in the *B. vulgaris* L./β-cyclodextrin complex was identified by means of HPLC analysis, according to the previous study (Figure 1) [32].

**Figure 2:**
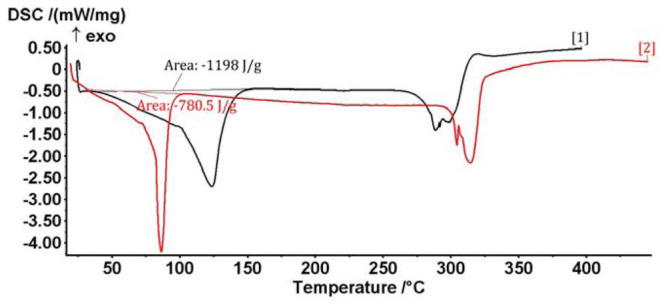
Differential scanning calorimetry (DSC) analysis of the *Berberis vulgaris* L. extract/β-cyclodextrin complex (red curve) and the corresponding β-cyclodextrin sample (black curve).

**Figure 3:**
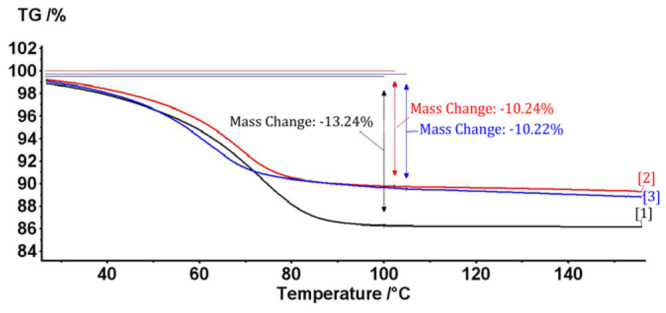
Thermogravimetry (TG) analysis of the *Berberis vulgaris* L. extract/β-cyclodextrin complexes (upper curves) and the corresponding β-cyclodextrin sample (lower curve).

The *B. vulgaris* L. extract/β-cyclodextrin complex formation is proved by thermal and calorimetric analyses. Thus, the overall profile of the DSC (differential scanning calorimetry) and TG (thermogravimetry) analyses of the complex are very different in comparison with the β-cyclodextrin case; the formation of the complex is especially revealed by the shifting of the water dissociation calorimetric process to lower temperatures in the DSC analysis. This is explained by the replacing of these water molecules from β-cyclodextrin with the bioactive *B. vulgaris* compounds (such as berberine, which was previously identified by HPLC analysis) (Figure 2). Furthermore, the water concentration in the β-cyclodextrin complex is approximately 3% lower than in the case of initial β-cyclodextrin, which are revealed by both DSC and TG analyses (1200 J/g for *B.v.*/βCD complex and 780 J/g for βCD in the DSC analysis; 10.2% mass loss for *B.v.*/βCD complex and 13.2% for βCD in the TG analysis; Figures 2 and 3). These means that some water molecules (especially from the β-cyclodextrin cavity) are replaced by *Berberis vulgaris* L. extract compounds (especially by the most concentrated berberine, revealed by HPLC analysis) in the nanoencapsulation process, which leads to the formation of *Berberis vulgaris* extract/β-cyclodextrin complex.

### 2.2. Effects of Berberis vulgaris Extract/β Cyclodextrin Pre-Treatment on Serum ALT, AST, γ-GT, Total and Direct Bilirubin

**Figure 4:**
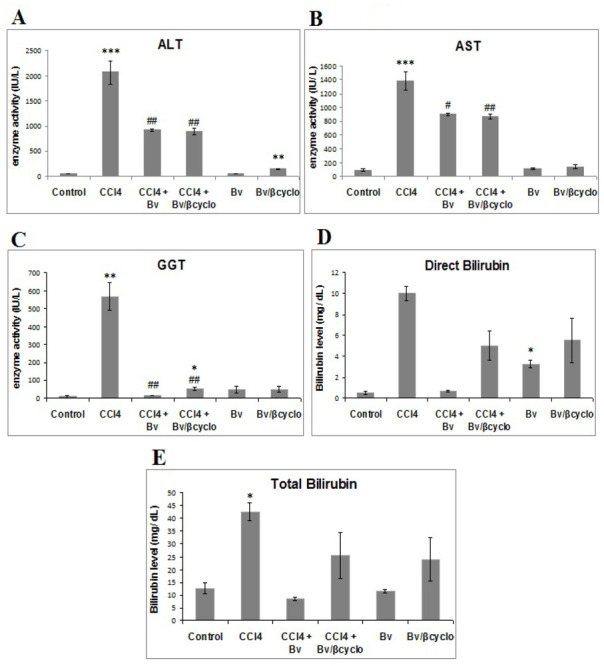
The effects of a 50 mg/kg dose of formulated and non-formulated *Berberis vulgaris* extract against liver injuries induced by CCl_4_ for 24 h on serum (**A**) alanine-amino-transferase (ALT); (**B**) apartate-amino-transferase (AST); (**C**) γ-glutamyl transferase (γ-GT); (**D**) direct bilirubin; (**E**) total bilirubin. Values are expressed as mean ± SD (*n* = 8). * *p* < 0.05 significantly different from the control group; ^#^ *p* < 0.05 significantly different from the CCl_4_-treated group.

Effects of non-formulated and formulated *Berberis vulgaris* extract on serum ALT, AST, γ-GT activities, as well as total and direct bilirubin, in mice from various treatment groups are shown in Figure 4. The dose of 50 mg/kg formulated and non-formulated extract of *Berberis vulgaris* was chosen according to previous experiments [31], taking into account that the maximum concentration of berberine was found in bark (the plant used for our extract preparation) followed by root, leaves and fruit [32].

After 24 h of CCl_4_ treatment, the ALT (Figure 4A), AST (Figure 4B) and γ-GT (Figure 4C) serum activities significantly increased (*p* < 0.001) by 40.46, 14.86 respectively 59.82 folds, whereas the total bilirubin and direct bilirubin levels significantly raised by 3.4 and 20 times compared to control group.

Pre-treatment with 50 mg/kg of *Berberis vulgaris* extract/β-cyclodextrin decreased significantly the CCl_4_-induced elevation of serum aminotransferases by 50% for ALT (Figure 4A). The non-formulated *Berberis vulgaris* extract showed no increase of ALT activity whereas the formulated one significantly raised it compared to control group. In the case of AST, both formulated and non-formulated *Berberis vulgaris* extracts reduced the activity elevation induced by CCl_4_ treatment by about 35%, whereas in the groups where only one type of extract was administered, AST levels were the same as in the control group (Figure 4B).

In addition, the up-regulation of γ-GT activity determined by CCl_4_ exposure was diminished by about 10 times by pre-treatment with formulated and non-formulated extracts (Figure 4C).

In the same time, a significant decrease of serum total and direct bilirubin by about 50% was observed in the pre-treated mice with formulated and non-formulated *Berberis vulgaris* extracts.

It is well known that chemical agents produce liver damage, causing high increases in bilirubin and enzymes activity presented above, which are released into serum [4,33,34]. The elevated activities of ALT, AST and γ-GT as well as total and direct bilirubin are indicative for cellular leakage and loss of the functional integrity of the liver cell membrane [35,36], and mitochondrial disruption respectively [37].

In our study, the pre-treatment with both formulated and non-formulated extracts, suppressed the increment of these parameters induced by CCl_4_ administration, results similar with those obtained by 160 mg/kg berberine pre-treatment [28]. It seems that the *Berberis vulgaris* extract entrapped in β cyclodextrin was more efficient at a lower dose than the alkaloid itself.

### 2.3. Effects of *Berberis vulgaris* Extract/β Cyclodextrin Pre-Treatment on Antioxidant Status and Lipid Peroxidation

**Figure 5:**
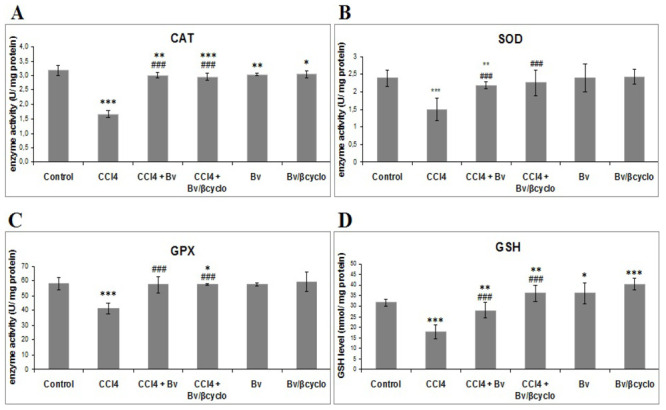
Protective effect of a 50 mg/kg dose of non-formulated *Berberis vulgaris* extract and *Berberis vulgaris* extract/βcyclodextrin on CCl_4_-induced oxidative stress in mice liver. Oxidative stress was assessed by measuring the (**A**) catalase **(**CAT); (**B**) superoxide dismutase (SOD) and (**C**) glutathione peroxidase (GP_X_) activity; (**D**) glutathione (GSH) level. * *p* < 0.05 significantly different from the control group; ^#^ *p* < 0.05 significantly different from the CCl_4_-treated group.

As shown in Figure 5, activities of CAT, SOD and GPx were significantly decreased in CCl_4_ treated mice compared to control ones. Formulated and non-formulated *Berberis vulgaris* extract pre-treatment significantly prevented inhibition of these enzymes activities caused by CCl_4_, and SOD activity of non-formulated *Berberis vulgaris* extract pre-treatment was slightly decreased compared to formulated extract and CCl_4_ treated group. In addition, CCl_4_ toxicity was confirmed by depletion of cellular GSH content in liver cells. Pre-treatment with non-formulated and *Berberis vulgaris*/β cyclodextrin extract determined a significant increase of GSH concentration compared to CCl_4_ groups. It should be mentioned that CAT, SOD and GP_X_ activities in mice liver treated only with formulated or non-formulated *Berberis vulgaris* extract were approximately equal to those from control group, and GSH content was higher in these two groups compared to control. The level of this tripeptide, important in redox homeostasis and free radical scavenging detoxification of electrophils, increased probably by stimulation of γ-glutamyl cycle and/or enzymatic regeneration of GSH from oxidized glutathione.

**Figure 6:**
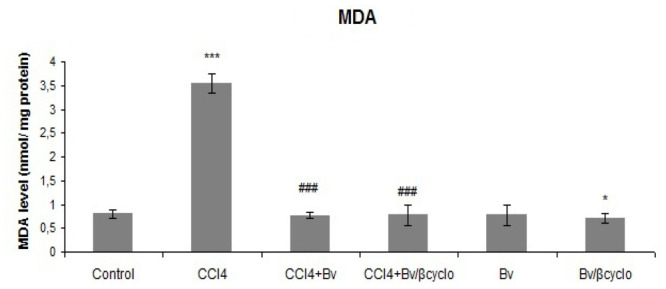
Protective effect of a 50 mg/kg dose of non-formulated *Berberis vulgaris* extract and *Berberis vulgaris* extract/βcyclodextrin CCl_4_-induced lipid peroxidation in mice liver. * *p* < 0.05 significantly different from the control group; ^#^ *p* < 0.05 significantly different from the CCl_4_-treated group.

Lipid peroxidation was determined by measuring the concentration of malondialdehyde. As Figure 6 illustrates, after 24 h of CCl_4_ treatment, MDA content significantly increased, but its high level was markedly reduced through treatment with formulated and non-formulated *Berberis vulgaris* extracts to levels almost similar to control group. In the case of treatment with *Berberis vulgaris* extracts alone, MDA levels close to normal group were observed.

Previous studies showed that CCl_4_ caused hepatocellular damages through an increase in the formation of DNA strand breaks, 8-oxodeoxiguanozine and MDA-DNA adducts under conditions of increased lipid peroxidation, and high depletion in glutathione content [38].

It is well known that antioxidant enzymes and GSH deficiency enhances the possibility for lipid peroxidation, and may cause irreversible damage to the liver tissue [39]. The protective effects of *Berberis vulgaris* extracts on the liver of CCl_4_ treated mice highlighted their elevated antioxidant activity, polyphenolic content, and rich nutritional composition, which sustain redox properties of extracts [40–42]. The antioxidant effects of *Berberis vulgaris* extract shown here were in agreement with a previous report [31].

We have shown that GPx and GSH levels of non-formulated extract pre-treatment group were statistically similar with those measured in the liver of *Berberis vulgaris* extract/β-cyclodextrin pre-treated mice. The pre-treatment with both *Berberis vulgaris* extracts attenuated CCl_4_ toxicity in a very efficient way, improving the antioxidant status of liver tissue.

It seems that they did not stimulate the antioxidant enzymes because CAT, SOD and GPx activities were not increased in mice treated only with formulated or non-formulated *Berberis vulgaris* extract.

### 2.4. Histopathology

**Figure 7:**
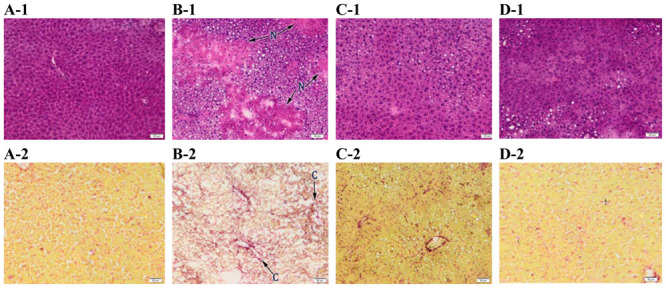

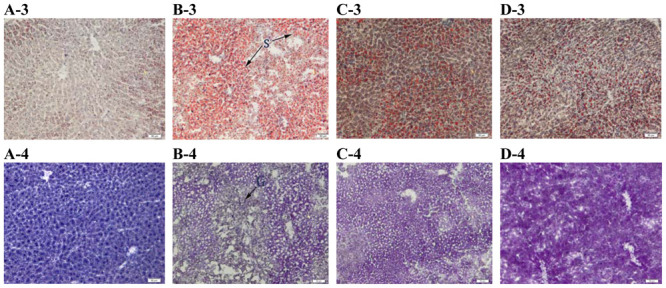
Protective effect of a 50 mg/kg dose of *Berberis vulgaris* extract and *Berberis vulgaris* extract/βcyclodextrin on structure of hepatocytes against liver injuries induced by CCl_4_. (**A**) Control group; (**B**) CCl_4_ group; (**C**) *Berberis vulgaris* extract + CCl_4_ group; (**D**) *Berberis vulgaris* extract/β cyclodextrin + CCl_4_ group. **1**. H & E staining (nuclei-blue; cytoplasm-red), **2**. Fouchet Van Gieson staining (nuclei-red; cytoplasm-yellow; collagen- red), **3**. Oil Red staining (nuclei-blue; lipid drops–red), **4**. P.A.S Periodic Acid Schiff staining (glycogen deposits—pink violet). N: centrilobular necrosis; C: collagen proliferation; S: steatosis; G: glycogen deposits depletion. Scale: 50 μm.

Light microscopic evaluation of liver tissues showed normal liver architecture (Figure 7A-1) without steatosis (Figure 7A-3) and fibrosis aspects (Figure 7A-2) in control group. Normal glycogen deposits are present into hepatocytes cytoplasm (Figure 7A-4).

After 24 h of CCl_4_ exposure, necrobiotic changes of hepatocytes including vacuolar degeneration, nuclear pyknosis and necrosis (Figure 7B-1) were observed, which were in agreement with previous reports [36,43]. The necrosis was more pronounced in the centrilobular area with the formation of bridges from one central vein to another (Figure 7B-1), followed by infiltration of inflammatory cells, sinusoid congestion, macro and microvesicular steatosis of hepatocytes (Figure 7B-3). Inflammatory cell infiltration and slight fibrosis around central vein and sinusoids were detected (Figure 7B-2). Glycogen deposits were severely diminished compared to the control group (Figure 7B-4), probably due to the high-energy demand of the hepatocytes for the possible repair processes in CCl_4_-given animals [44].

The seven-day preventive treatment with 50 mg/kg of formulated and non-formulated *Berberis vulgaris* extract, showed mild to moderate accumulation of lipid drops and reduction of inflammatory infiltrates compared to the CCl_4_ group (Figure 7C-3, D-3). In both *Berberis* pre-treated groups, an absence of hepatocellular necrosis areas (Figure 7C-1, D-1) was observed. Vacuolated hepatocytes were seen in some centrilobular areas but to a lesser extent compared to CCl_4_ group (Figure 7C-1, D-1). Similarly, Feng *et al.* [28] reported that fatty content change, necrosis and lymphocyte infiltration were improved in the histological sections from 80, 12 and 160 mg/kg berberine pre-treated and post-treated mice in a dose-dependent manner.

Fibrosis around central vein and sinusoids was reduced in both pre-treated groups (Figure 7C-2, D-2). Similar results were observed with berberine that prevented liver fibrosis due to the decreased number of hepatic stellate cells (HSCs) [45] or by regulating the lipid peroxidation and antioxidant system [46].

Also, glycogen deposits were highly restored in the *Berberis vug*aris extract/β-cyclodextrin group (Figure 7D-4) than in non-formulated *Berberis vulgaris* extract pre-treatment (Figure 7C-4). Our results are in accordance with previous data which have shown that hepatic glycogen levels increased after 75 mg/kg of berberine were administered to rats [47].

### 2.5. Electron Microscopy

**Figure 8:**
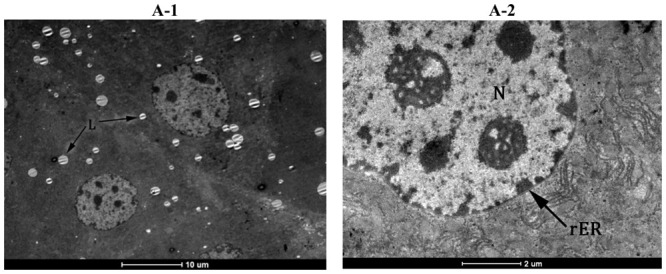

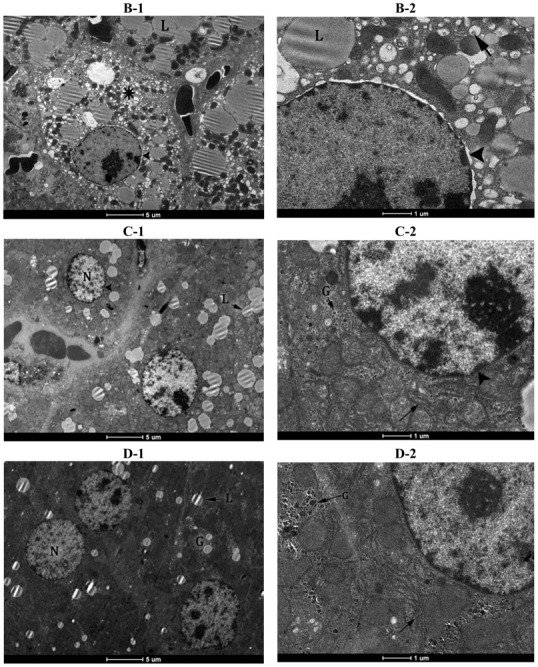
Protective effect of a 50 mg/kg dose of *Berberis vulgaris* extract and *Berberis vulgaris* extract/βcyclodextrin on ultrastructure of hepatocytes against liver injuries induced by CCl4. (**A**) Control group; normal aspect of nucleus (N), glycogen deposits (G) and few lipid drops (L); (**B**) CCl_4_ group; oedematous cytoplasm matrix with sER proliferation (asterix); dilated rER profiles (arrow) and enlarged nuclear space (arrowhead); increased number and size of lipid drops (L); (**C**) *Berberis* extract + CCl_4_ group; normal aspect of nuclear shape (arrowhead) and rER (arrow);Mild reduction of number and size of lipid drops (L) and moderate recovery of glycogen deposits (G); (**D**) *Berberis vulgaris* extract/βcyclodextrin + CCl_4_ group; Glycogen deposits recovery (G) and normal aspect of rER (arrow).

The ultrastructure of hepatocytes was normal in the control group with regular aspect of nuclear shape and rER’s profiles (Figure 8A-2), few lipid globules, and normal glycogen deposits into cytoplasm (Figure 8A-1). In the CCl_4_ treated group most of the hepatocytes showed large lipid globules and glycogen loss, according to optic microscopy analysis. Some hepatocytes showed an oedematous cytoplasm matrix with two populations of lipid globules -large and small, degenerated organelles and proliferation of smooth reticulum vesicles (Figure 8B-1). There were irregular lamellar organization and large dilatations with focal breaks in rERs of hepatocytes of CCl_4_ treated group in many areas, associated with dilated perinuclear space and enlarged pores (Figure 8B-2). The changes of hepatocytes ultrastructure [48] were probably due to the injuries in membrane structure caused by lipid peroxidation.

Pre-treatment with 50 mg/kg of formulated and non-formulated *Berberis vulgaris* extract significantly reduced the volume and number of lipid globules in the hepatocytes (Figure 8C-1, D-1), more obvious than in non-formulated *Berberis vulgaris* extract (Figure 8C-1). The organelle and cytoplasm structures were widely protected against the effects of CCl_4_. Large dilatations with focal breaks in rERs of hepatocytes, associated with dilated perinuclear space and enlarged pores were not seen in either pre-treated group (Figure 8C-2, D-2). Although glycogen deposits are increased both in formulated and non-formulated *Berberis vulgaris* groups compared to CCl_4_ (Figure 8C-2, D-2).

### 2.6. Effects of *Berberis vulgaris* Extract/β-Cyclodextrin Pre-Treatment on DNA Damage

**Figure 9:**
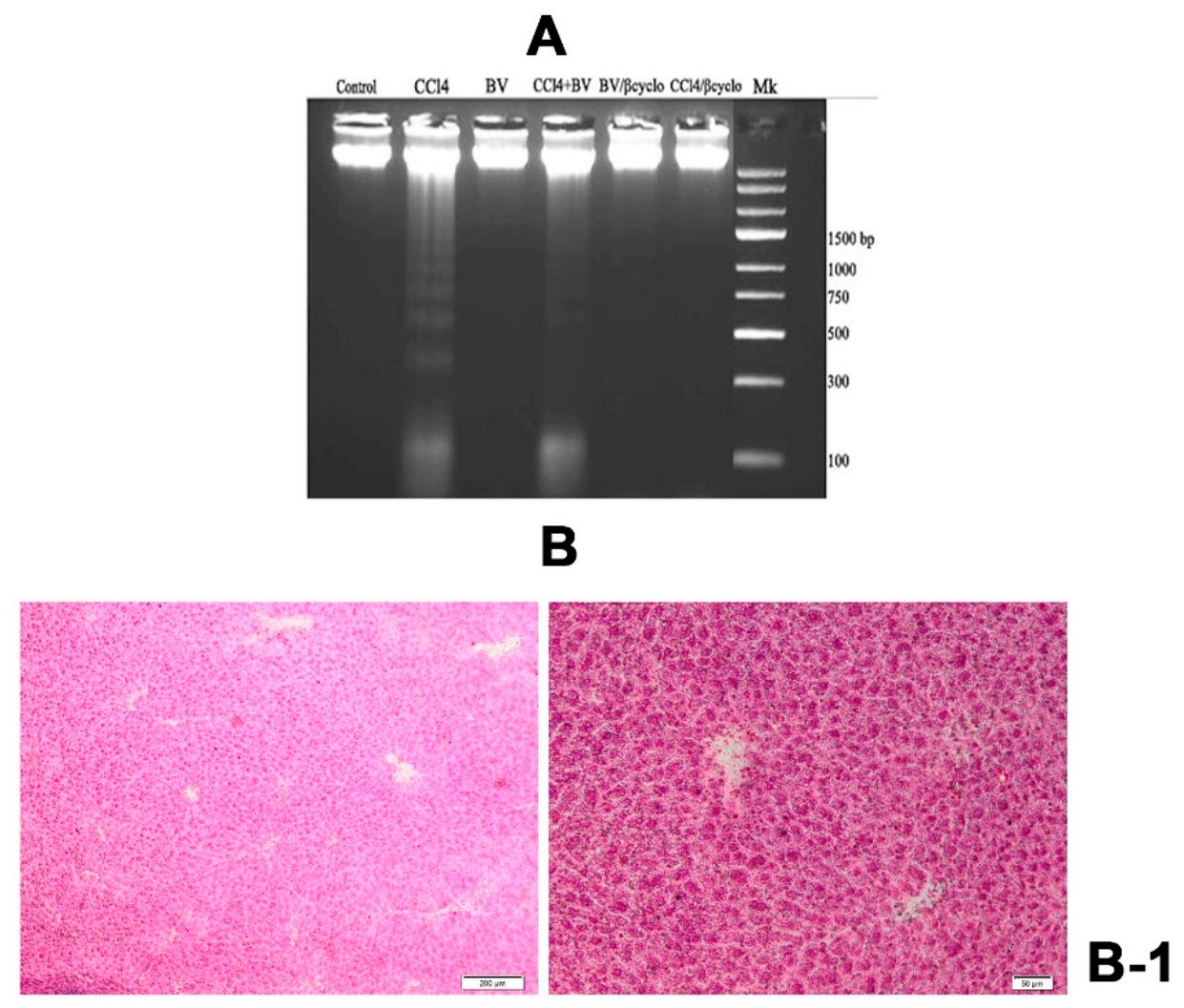

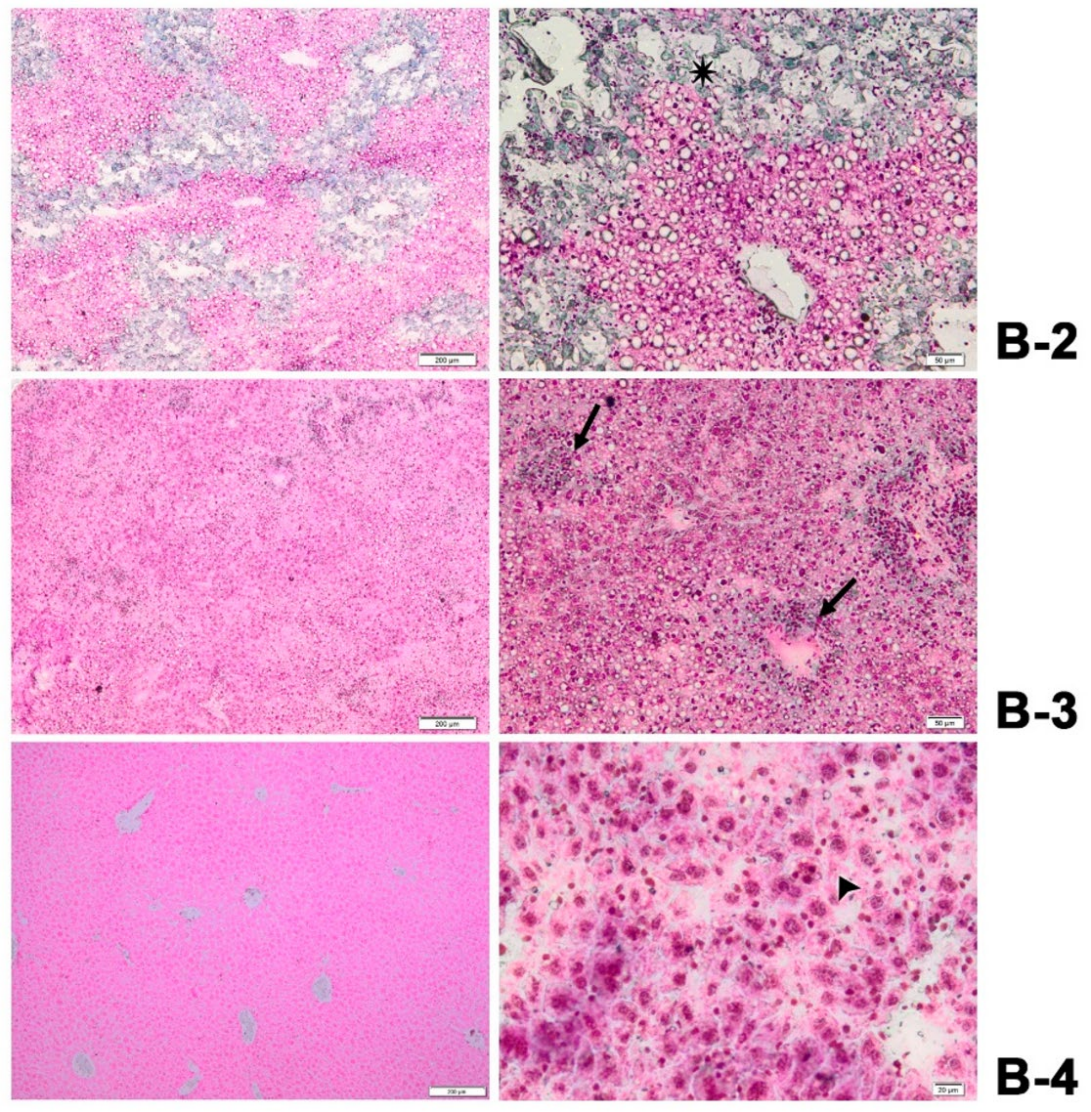
Effects of *Berberis vulgaris* extract/β-cyclodextrin on CCl_4_-induced DNA damage. (**A**) Lane 1-DNA isolated from normal liver; Lane 2: DNA isolated from CCl_4_ intoxicated liver; Lane 3: DNA isolated from liver treated with *Berberis vulgaris*; Lane 4: DNA isolated from liver pre-treated with *Berberis vulgaris* extract followed by i.p. CCl_4_ injection; Lane 5: DNA isolated from liver treated with *Berberis vulgaris* extract/β cyclodextrin; Lane 6: DNA isolated from liver pre-treated with *Berberis vulgaris* extract/β cyclodextrin followed by i.p. CCl_4_ injection; Lane 7: Marker (1-kb DNA ladder). (**B**) Methyl-green pyronin staining of liver. (**B-1**): Control group; (**B-2**): CCl_4_ group; extended ssDNA areas (asterix); (**B-3**): *Berberis vulgaris* extract + CCl_4_; semnificative reduction of ssDNA areas (arrow); (**B-4**): *Berberis vulgaris* extract/β cyclodextrin + CCl_4_; ssDNA areas loss and normal RNA distribution (arrowhead) (DNA—pale green; RNA—pink).

DNA internucleosomal fragmentation was seen in liver samples of CCl_4_ treated group and a reduced level of it appeared in the mice group pre-treated with non-formulated *Berberis vulgaris* extract. At the same time, the pre-treatment with *Berberis vulgaris* extract/β-cyclodextrin completely suppressed this process (Figure 9A). Also, no DNA internucleosomal fragmentation was induced by either formulated and non-formulated *Berberis vulgaris* extracts in the absence of CCl_4_ treatment.

The methyl-green pyronin (MGP) histochemical investigation of liver tissue was in agreement with DNA fragmentation assay. Large pale green areas of damaged DNA were seen in the CCl_4_ group especially in the centrilobular area with the formation of bridges from one central vein to another (Figure 9B-2) which overlap with areas of the liver affected by necrobiosis observed in H & E micrographs. These areas are more reduced in the group pre-treated with non-formulated *Berberis vulgaris* extract (Figure 9B-3) and are absent in the group pre-treated with *Berberis vulgaris* extract/β-cyclodextrin (Figure 9B-4), as in the case of normal mice, where intense pink areas of RNA are also present (Figure 9B-1).

DNA strand breaks are a cause of direct modification of DNA by chemical agents or their metabolites, which occurs when reactive oxygen species (ROS) interact with DNA [49,50]. In our study, the type of cell death induced by CCl_4_ was investigated by DNA fragmentation assay and methyl-green pyronin (MGP) histochemical staining, which highlights the presence of RNA and ssDNA into liver tissue [51–53]. Internucleosomal DNA fragmentation was observed in liver samples of 1.0 mL/kg CCl_4_ intoxicated group, which is the indication that cells were undergoing apoptotic or necrotic death as in previous studies [54–56]. The absence of DNA ladder and of MGP staining in the liver of mice pretreated with *Berberis vulgaris* extract/β-cyclodextrin and intoxicated with CCl_4_ indicated the hepatoprotective role of the formulated extract. The non-formulated extract counteracted the damaging effect of CCl_4_ but to a lesser extent, possibly due to the increased bioavilability of β cyclodextrin formulation as already it was proved in the case of liver injuries [57–59].

## 3. Experimental Section

### 3.1. *Berberis vulgaris* Extract/β-Cyclodextrin Formulation

Samples of *Berberis vulgaris* L. were collected from the Botanical Garden of Vasile Goldis Western University of Arad during October 2008 and certified at the herbarium in the Department of Botany, Faculty of Natural Sciences, where a voucher specimen already exist. The bark of *Berberis vulgaris* L. was separated and frozen until extraction. Bioactive compounds from *Berberis vulgaris* samples were separated by solid-liquid extraction in 96% ethanol, according to the method described by Hadaruga *et al* [32]. The final extract was subjected to HPLC analysis [32] followed by β-cyclodextrin nanoencapsulation [32,60]. The evaluation of *Berberis vulgaris*/β-cylodextrin complex formation was proved by differential scanning calorimetry (DSC) and thermogravimetry (TG) according to the previous study [32]. The dried form of the formulated and non-formulated extract*,* dissolved in distilled water was administered orally (50 mg/kg b.w.) to mice so as to check hepatoprotective activities.

### 3.2. Animals and Experimental Procedure

Adult male Swiss mice weighing 25 ± 3 g were obtained from Animal House of the Vasile Goldis Western University of Arad. The animals were left for 2 days for acclimatization to animal room conditions maintained on standard pellet diet and water *ad libitum* at a temperature of 20–25 °C under a 12 h light/dark cycle throughout the experiment. The food was withdrawn on the day before the sacrifice. All animals received human care and study protocols complied with the guidelines of the Animal House. Throughout the experiments, animals were processed according to the international ethical guidelines for the care of laboratory animals.

Forty-eight animals were divided into 6 groups, as follows: group 1 (control group), group 2 (CCl_4_ treated group), group 3 (pre-treated with 50 mg/kg b.w. of *Berberis vulgaris* extract and subsequently intraperitoneally injected with CCl_4_), group 4 (pre-treated with 50 mg/kg b.w. of *Berberis vulgaris* extract/β-cyclodextrin and subsequently i.p. injected with CCl_4_), group 5 (treated with 50 mg/kg b.w. of *Berberis vulgaris* extract), group 6 (treated with 50 mg/kg b.w. of *Berberis vulgaris* extract/β-cyclodextrin). Non-formulated *Berberis vulgaris* extract and *Berberis vulgaris* extract/β-cyclodextrin complex were orally administrated to mice from groups 3 and 5 and to groups 4 and 6 for 7 days. Mice from groups 2, 3, and 4 were intraperitoneally (i.p.) injected with CCl_4_ at a dose of 1.0 mL/kg body weight in 50% olive oil (1:1) on the 8th day. Control and CCl_4_ treated groups received the equivalent volume of distilled water orally for 7 days.

24 h after CCl_4_ i.p. injection the mice were sacrificed by cervical dislocation. Serum and tissue samples were used for histopathology, electron microscopy and biochemical analyses.

### 3.3. Animals and Experimental Procedure

Homogenates (prepared as 1 g of tissue per 10 volumes of buffer) of mice livers were prepared in ice-cold buffer (0.1 M TRIS-HCl, 5 mM EDTA buffer, pH 7.4) and homogenized for 2 min at 16 Hz using a ball mill (type MM 301, Retsch GmbH & Co, Haan, Germany). The homogenate was centrifuged at 10,000 rpm for 30 min in a Hettich centrifuge at 4 °C to remove the cell debris A few crystals of the protease inhibitor phenylmethylsulfonyl fluoride were added in the homogenates. The supernatant was decanted and used for biochemical assays.

Blood was collected from retro-orbital venous plexus. The tubes with whole blood were incubated for 60 min at room temperature to allow clotting. They were then centrifuged for 15 min at 2000 rpm. The supernatant (serum) was aliquoted and used immediately for biochemical analyses.

### 3.4. Histopathology

Frozen liver sections were cut at 8 μm at SLEE MNT cryotome, fixed in 10% buffered formaldehide and stained with hematoxylin-eosin, oil red, P.A.S. periodic acid Schiff, Fouchet van Gieson and methyl-green pyronin, according to the methods of Bio-Optica staining kits. Mounted slides were examined under a light microscope (Olympus BX43 microscope) and photographed using a digital camera Olympus XC30.

### 3.5. Electron Microscopy

For ultrastructural investigations, liver samples were prefixed in 2.7% glutaraldehyde solution in 0.1 M phosphate buffer for 1.5 h, at 4 °C. Then they were washed in 0.15 M phosphate buffer (pH 7.2). Postfixation was performed in 2% osmic acid solution in 0.15 M phosphate buffer for 1 h at 4 °C. Dehydration was performed in acetone, and inclusion in the epoxy embedding resin Epon 812. The blocks have been cut at an ultramicrotome type LKB, at 70 nm thickness. The sections were doubly contrasted with solutions of uranyl acetate and lead citrate and were analyzed with TEM Tecnai 12 Biotwin electron microscope.

### 3.6. Biochemical Assays

#### 3.6.1. Activities of Serum Hepatic Markers

The serum activities of aspartate aminotransferase (AST), alanine aminotransferase (ALT), γ-glutamyl transferase (γ-GT), direct and total bilirubin were evaluated by the spectrophotometric method using commercially available kits (Roche reagents, Meylan, France) according to the manufacturer’s instructions.

#### 3.6.2. Assesment of Antioxidant Status

Hepatic catalase (CAT) activity was determined by the Aebi method [61] which records the decomposition of H_2_O_2_ by decrease in the absorbance at 240 nm. One unit of CAT activity is equal to the decomposition of one μmole H_2_O_2_/min/mL. Liver superoxide dismutase (SOD) activity was measured by the method described by Beauchamp and Fridovich [62]. One unit of SOD activity is defined as the amount of enzyme that inhibits the oxidation of NADH by 50% at 37 °C. The measurement of GPx activity was performed according to the method of Beutler *et al.* [63] which is based on monitoring of the oxidation of NADPH at 340 nm (V-530 JASCO spectrophotometer). All enzymatic activities, calculated as specific activities (units/mg of protein) were expressed as % from controls.

GSH content was measured with a commercial kit (Sigma-Aldrich, Taufkirchen, Germany) according to the manufacturer’s instructions. The absorbance was measured at 405 nm using a microtiter plate reader (GENIOS Tecan) and the concentration was expressed in nmoles GSH/mg protein.

#### 3.6.3. Assay of Lipid Peroxidation

The hepatic malondialdehyde (MDA) content, a measure of lipid peroxidation, was assayed using a fluorimetric method described by Del Rio *et al.* [64]. Briefly, the sample (200 μL) was incubated with 0.1 M HCl (700 μL) for 20 min at room temperature. A volume of 900 μL of 0.025 M thiobarbituric acid was added, and the mixture was maintained at 37 °C for 65 min. Then the samples were subjected to fluorescence analysis (λ_ex_ = 520 nm; λ_em_ = 549 nm) (Spectrofluorometer FP-6300 JASCO). Relative fluorescence units (RFU) were converted to nmoles malondialdehyde (MDA) using 1,1,3,3-tetramethoxypropane as standard.

#### 3.6.4. Protein Concentration Measurement

The protein content was determined using Lowry’s method with bovine serum albumine as standard [65].

### 3.7. DNA Fragmentation Assays

Following the method of Zhou *et al.* [66], 0.15 g of frozen liver was homogenized in 1.5 mL lysis buffer [10 mM Tris-HCl pH 7.5; 100 mM EDTA; 0.5% SDS; 0.1 mg/L proteinase K]. RNA-free DNA from each sample was extracted twice with phenol, once with phenol-chloroform-isoamyl alcohol (PCI 25:24:1) and once with chloroform. After centrifugation, DNA was precipitated with cold ethanol and sodium acetate. The pellet was dissolved in TE buffer (10 mM Tris-HCl; 1.0 mM EDTA) and incubated with RNase (0.1 mg/L) at 37 °C for 45 min. Subsequently the suspension was re-extracted with PCI and chloroform and precipitated as described above. A quantity of 3 μg of DNA was loaded on 1.5% agarose gel and run at 100 V. The gel was stained with ethidium bromide and photographed under UV light using UVP BioDoc-IT Imaging System.

### 3.8. Statistical Analysis

All results were expresed as mean ± SEM. The data were analyzed for statistical significance using Student’s *t* test. A value of *p* < 0.05 was considered significant: *; distinct significant at *p* < 0.01: **; very significant at *p* < 0.001: ***.

## 4. Conclusions

In conclusion, this study showed that *Berberis vulgaris* extract/β-cyclodextrin presented better hepatoprotective effects than free extract on oral administration probably due to its increased bioavailability. It seems that formulated extract could be used as a low cost phytotherapeutical supplement suitable for acute or chronic conditions of liver diseases or as a supportive treatment in addition to conventional therapies of serious hepatic diseases. Taken together, our results suggest that *Berberis vulgaris* extract/β-cyclodextrin should be further investigated regarding clinical applications.

## References

[1] Jones A.L., Zakin D., Boyer T.D. (1996). Anatomy of the Normal Liver. Hepatology: A Textbook of Liver Disease.

[2] Zimmerman H.J. (1978). Hepatotoxicity: The Adverse Effects of Drugs and other Chemicals on the Liver.

[3] Kaminski M., Wiaderkiewicz R. (2007). The role of the liver in xenobiotic biotransformation. Part I. The role of the liver and its cells and their interactions. Probl. Forensic Sci.

[4] Weber L.W., Boll M., Stampfl A. (2003). Hepatotoxicity and mechanism of action of haloalkanes: Carbon tetrachloride as a toxicological model. Crit. Rev. Toxicol.

[5] Merrick B.A. (2006). Toxicoproteomics in liver injury and inflammation. Ann. N. Y. Acad. Sci.

[6] Manibusan M.K., Odin M., Eastmond D.A. (2007). Postulated carbon tetrachloride mode of action: A review. J. Environ. Sci. Health C.

[7] Luper S. (1998). A review of plants used in the treatment of liver disease: Part 1. Altern. Med. Rev.

[8] Luper S. (1999). A review of plants used in the treatment of liver disease: Part 2. Altern. Med. Rev.

[9] Murriel P., Rivera-Espinoza Y. (2008). Beneficial drugs for liver diseases. J. Appl. Toxicol.

[10] Ivanovska N., Philipov S. (1996). Study on the anti-inflamatory action of *Berberis vulgaris* root extract, alkaloid fractions and pure alkaloids. Int. J. Immunopharmacol.

[11] Saied S., Begum S. (2004). Phytochemical studies of *Berberis vulgaris*. Chem. Nat. Compd.

[12] Imanshahidi M., Hosseinzadeh H. (2008). Pharmacological and therapeutical effects of *Berberis vulgaris* and its active constituent, berberine. Phytother. Res.

[13] Freile M.L., Giannini F., Pucci G. (2003). Antimicrobial activity of aqueolus extracts and of berberine isolates from *Berberis heterophylla*. Fitoterapia.

[14] Mahady G.B., Pendland S.L., Stoia A., Chaadwick L.R. (2003). *In vitro* susceptibility of *Helicobacter pylori* to isoquinoline alkaloids from *Sanguinaria canadensis* and *Hydrastis canadensis*. Phytother. Res.

[15] Iizuka N., Miyamoto K., Okita K., Tangoku A., Hayashi H., Yosino S. (2000). Inhibitory effect of Coptidis rhizoma and berberine on the proliferation of human esophageal cancer cell lines. Cancer Lett.

[16] Thirupurasundari C.J., Padmini R., Devaraj S.N. (2009). Effect of berberine on the antioxidant status, ultrastructural modifications and protein bound carbohydrates in azoxymethane-induced colon cancer in rats. Chem. Biol. Interact.

[17] Wang N., Feng Y., Zhu M., Tsang C.M., Man K., Tong Y., Tsao S.W. (2010). Berberine induces autophagic cell death and mitochondrial apoptosis in liver cancer cells: The cellular mechanism. J. Cell. Biochem.

[18] Kuo C.L., Chi C.W., Liu T.Y. (2004). The anti-inflamatory potential of berberine *in vitro* and *in vivo*. Cancer Lett.

[19] Kupeli E., Kosar M., Yesilada E., Husnu K., Baser C. (2002). A comparative study on the anti-inflamatory, antinociceptive and antipyretic effects of isoquinoline alkaloids from the root of Turkish *Berberis* species. Life Sci.

[20] Singh A., Duggal S., Kaur N., Singh J. (2010). Berberine: Alkaloid with wide spectrum of pharmacological activities. J. Nat. Prod.

[21] Hobara N., Watanabe A. (1984). Berberine-Induced bile bilirubin secretion in the rat. Curr. Ther. Res. Clin. Exp.

[22] Rabbani G.H., Butler T., Knight J., Sanyal S.C., Alam K. (1978). Randomized controlled trial of berberine sulfate therapy for diarrhea due to enterotoxigenic *Escherichia coli* and *Vibrio cholera*. J. Infect. Dis.

[23] Zhou H., Mineshita S. (2000). The effect of berberine chloride on experimental cholitis in rats *in vivo* and *in vitro*. J. Pharmachol. Exp. Ther.

[24] Fatehi M., Saleh T.M., Fatehi-Hassanabad Z., Farrokhfal K., Jafarzadeh M., Davodi S. (2005). A pharmacological study on *Berberis vulgaris* fruit extract. J. Ethnopharmacol.

[25] Wong K.K. (1998). Mechanism of the aortic relaxation induced by low concentrations of berberine. Planta Med.

[26] Zeng X.H., Zeng X.J., Li Y.Y. (2003). Efficacy and safety of berberine for congestive heart failure secondary to ischemic or idiopathic dilated cardiomyophathy. Am. J. Cardiol.

[27] Lee B., Yang C.H., Hahm D.H., Choe E.S., Lee H.J., Pyun K.H., Shim I. (2009). Inhibitory Effects of *Coptidis rhizoma* and Berberine on Cocaine-induced Sensitization. Evid. Complement. Altern. Med.

[28] Feng Y., Siu K.Y., Ye X., Wang N., Yuen M.F., Leung C.H., Tong Y., Kobayashi S. (2010). Hepatoprotective effects of berberine on carbon tetrachloride-induced acute hepatotoxicity in rats. Chin. Med.

[29] Janbaz K.H., Gilan A.H. (2000). Studies on preventive and curative effects of berberine on chemical-induced hepatotoxicity in rodents. Fitoterapia.

[30] Hwang J.M., Wang C.J., Chou F.P. (2002). Inhibitory effect of berberine on tert-butyl hydroperoxide-induced oxidative damage in rat liver. Arch. Toxicol.

[31] Fallah H., Zarrei M., Ziai M., Mehrazma M., Alavian S.M., Kianbakht S., Mehdizadeh M. (2010). The effects of *Taraxacum officinale* L. and *Berberis vulgaris* L. root extracts on carbon tetrachloride induced liver toxicity in rats. J. Med. Plants.

[32] Hadaruga D.I., Hadaruga N.G., Bandur G.N., Rivis A., Costescu C., Ordodi V.L., Ardelean A. (2010). *Berberis vulgaris* extract/β cyclodextrin nanoparticles synthesis and characterization. Rev. Chim. (Bucharest).

[33] Chaung S.S., Lin C.C., Lin J., Yu K.H., Hsu Y.F., Yen M.H. (2003). The hepatoprotective effects of *Limonium sinense* against carbon tetrachloride and beta-D-galactosamine intoxication in rats. Phytother. Res.

[34] Clawson G.A. (1989). Mechanism of carbon tetrachloride hepatotoxicity. Pathol. Immunopathol. Res.

[35] Bhadauria M (2012). Propolis prevents hepatorenal injury induced by chronic exposure to carbon tetrachloride. Evid. Complement. Altern. Med.

[36] Shaker E. (2010). Sylimarin, the antioxidant component and *Silybum marianum* extracts prevent liver damage. Food Chem. Toxicol.

[37] Xu L., Gao J., Wang Y., Yu W., Zhao X., Yang X., Zhong Z., Qian Z.M. (2011). *Myrica rubra* extracts protect the liver from CCl_4_-induced damage. Evid. Complement. Altern. Med.

[38] Beddowes E.J., Faux S.P., Chipman J.K. (2003). Chloroform, carbon tetrachloride and glutathione depletion induce secondary genotoxicity in liver cells via oxidative stress. Toxicology.

[39] Kohen R., Nyska A. (2010). Oxidation of biological systems: Oxidative stress phenomena, antioxidants, redox reactions, and methods for their quantification. Toxicol. Pathol.

[40] Motalleb G., Hanachi P., Kua S.H., Fauziah O., Asmah R. (2005). Evaluation of phenolic content and total antioxidant activity in *Berberis vulgaris* fruit extract. J. Biol. Sci.

[41] Parichehr H. (2009). Using HPLC to determination the composition and antioxidant activity of *Berberis vulgaris*. Eur. J. Sci. Res.

[42] Zovko M.K., Kremer D., Karlović K., Kosalec I. (2010). Evaluation of antioxidant activities and phenolic content of *Berberis vulgaris* L. and *Berberis croatica* Horvat. Food Chem. Toxicol.

[43] Ozturk F., Gul G., Ates B., Ozturk I.C., Cetin A., Vardi N., Otlu A., Yilmaz I. (2009). Protective effect of apricot (*Prunus armeniaca* L.) on hepatic steatosis and damage induced by carbon tetrachloride in Wistar rats. Br. J. Nutr.

[44] Junnila M., Rahko T., Sukura A. (2000). Reduction of carbon tetrachloride-induced hepatotoxic effects by oral administration of betaine in male Han-Wistar rats: A morphometric histological study. Vet. Pathol.

[45] Sun X., Zhang X., Hu H., Lu Y., Chen J., Yasuda K., Wang H. (2009). Berberine inhibits hepatic stellate cell proliferation and prevents experimental liver fibrosis. Biol. Pharm. Bull.

[46] Zhang Q., Xiao X., Feng K., Wang T., Li W., Yuan T., Sun X., Sun Q., Xiang H., Wang H (2011). Berberine moderates glucose and lipid metabolism through multipathway mechanism. Evid. Complement. Altern. Med.

[47] Zhou J.Y., Zhou S.W., Zhang K.B., Tang J.L., Guang L.X., Ying Y., Xu Y., Zhang L., Li D.D. (2008). Chronic effects of berberine on blood, liver glucolipid metabolism and liver PPARs expression in diabetic hyperlipidemic rats. Biol. Pharm. Bull.

[48] Tasci I., Mas N., Mas M.R., Tuncer M., Comert B. (2008). Ultrastructural changes in hepatocytes after taurine treatment in CCl_4_ induced liver injury. World Gastroenterol.

[49] Folkmann J.K., Risom L., Jacobsen N.R., Loft H.W.S., Moller P. (2009). Oxidatively damaged DNA in rats exposed by oral gavage to C60 fullerenes and single-walled carbon nanotubes. Environ. Health Perspect.

[50] Moller P., Wallin H. (1998). Adduct formation, mutagenesis and nucleotide excision repair of DNA damage produced by reactive oxygen species and lipid peroxidation product. Mutat. Res.

[51] Iseki S., Mori T. (1986). Methyl green pyronin stain distinguishes prolifferating from differentiated nonproliferating cell nuclei after acid denaturation of DNA. J. Histochem.

[52] Sen J.Y., Huang Q.Y., Gao H.Y., Liu Y.L., Cheng C.F. (1999). Modification and application of methyl green-pyronin stain after acid denaturation of DNA. Prog. Anat. Sci.

[53] Wang H.M., Zheng N.G., Wu J.L., Gong C.C., Wang Y.L. (2005). Dual effects of 8-Br-cAMP on differentiation and apoptosis of human esophageal cancer. World J. Gastroenterol.

[54] Manna P., Bhattacharyya S., Das J., Ghosh J., Parames C., Sil C (2011). Phytomedicinal role of *Pithecellobium dulce* against CCl_4_-mediated hepatic oxidative impairments and necrotic cell death. Evid. Complement. Altern. Med.

[55] Sakr S.A., El-Abd S.F., Osman M., Kandil A.M., Helmy M.S. (2011). Ameliorative effect of aqueous leave extract of *Ocimum basilicum* on CCl4-induced hepatotoxicity and apoptosis in albino rats. J. Am. Sci.

[56] Sengupta M., Sharma G.D., Chakraborty B. (2011). Effect of aqueous extract of *Tinospora cordifolia* on functions of peritoneal macrophages isolated from CCl4 intoxicated male albino mice. BMC Complement. Altern. Med.

[57] Linn T.T., Wang B.M., Li X.Y., Pan Y.W., Liu J.Q., Shen J.C., Luo G.M. (2009). An insight into the protection of rat liver against ischemia/reperfusion injury by 2-selenium-bridged beta-cyclodextrin. Hepatol. Res.

[58] Liu Y., Sakagami H., Hasimoto K., Kikuchi H., Amano O., Ishara M., Kanda Y., Kunii S., Kochi M., Zhang W., Yu G. (2008). Tumor-specific cytotoxicity and type of cell death induced by beta-cyclodextrin benzaldehyde inclusion compound. Anticancer Res.

[59] Yadav V.R., Prasad S., Kannappan R., Ravindran J., Chatuvedi M.M., Vaahtera L., Parkkinen J., Aggarwal B.B. (2010). Cyclodextrin-complexed cucurmin exhibits anti-inflamatory and antiproliferative activities superior to those of cucurmin through higher cellular uptake. Biochem. Pharmacol.

[60] Hadaruga D.I., Hadaruga N.G., Hermenean A., Rivis A., Paslaru V., Codina G. (2008). Biomaterials: Thermal stability of the oleic acid/α and β cyclodextrin complexes. Rev. Chim. (Bucharest).

[61] Aebi H, Bergmayer H.U. (1974). Catalase in Methods of Enzymatic Analysis.

[62] Beauchamp C.O., Fridovich I. (1971). Superoxide dismutase: Improved assays and an assay applicable to acrylamide gels. Anal. Biochem.

[63] Beutler E (1974). Red Cell Metabolism: A Manual of Biochemical Methods.

[64] Del Rio D., Pellegrini N., Colombi B., Bianchi M., Serafini M., Torta F., Tegoni F., Musci M., Brighenti F. (2003). Rapid fluorimetric method to detect total plasma malondialdehyde with mild derivatization conditions. Clin. Chem.

[65] Lowry O.H., Rosebrough N.J., Farr A.L., Randall R.J. (1951). Protein measurement with the folin phenol reagent. J. Biol. Chem.

[66] Zhou B.R., Gumenscheimer M., Freudenberg M., Galanos C. (2003). A sriking correaltion between lethal activity and apoptotic DNA fragmentation of liver in response of D-galactosamine-sintetized mice to a non-lethal amount of lipopolysaccharide. Acta Pharmacol. Sin.

