# Correction: Hermenean et al. Hepatoprotective Effects of *Berberis vulgaris* L. Extract/β Cyclodextrin on Carbon Tetrachloride–Induced Acute Toxicity in Mice. *Int. J. Mol. Sci.* 2012, *13*, 9014–9034

**DOI:** 10.3390/ijms27020788

**Published:** 2026-01-13

**Authors:** Anca Hermenean, Cristina Popescu, Aurel Ardelean, Miruna Stan, Nicoleta Hadaruga, Ciprian-Valentin Mihali, Marieta Costache, Anca Dinischiotu

**Affiliations:** 1 Department of Histology, Faculty of Medicine, Pharmacy and Dentistry, Vasile Goldis Western University of Arad, 1 Feleacului, 310396 Arad, Romania; anca.hermenean@gmail.com; 2 Department of Experimental and Applied Biology, Institute of Life Sciences, Vasile Goldis Western University of Arad, 86 Rebreanu, 310414 Arad, Romania; pursega36@gmail.com (C.P.); aardelean@uvvg.ro (A.A.); mihaliciprian@yahoo.com (C.-V.M.); 3 Department of Biochemistry and Molecular Biology, University of Bucharest, 91-95 Splaiul Independentei, 050095 Bucharest, Romania; miruna_stan@yahoo.com (M.S.); marietacostache@yahoo.com (M.C.); 4 Food Quality Department, Faculty of Food Processing Technology, Banat’s University of Agricultural Sciences and Veterinary Medicine, 119 Calea Aradului, 300645 Timisoara, Romania; nico_hadaruga@yahoo.com

In the original publication [1], there was a mistake in Figure 9 as published. The subfigures B-3 and B-4 were mistakenly selected, due to a human error during figure preparation. The corrected Figure 9 appears below. The authors state that the scientific conclusions are unaffected. This correction was approved by the Academic Editor. The original publication has also been updated.

**Figure 9:**
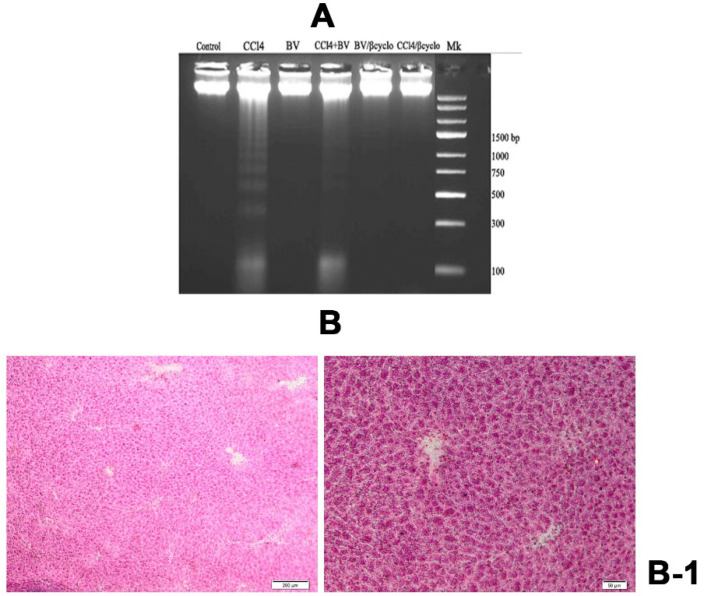

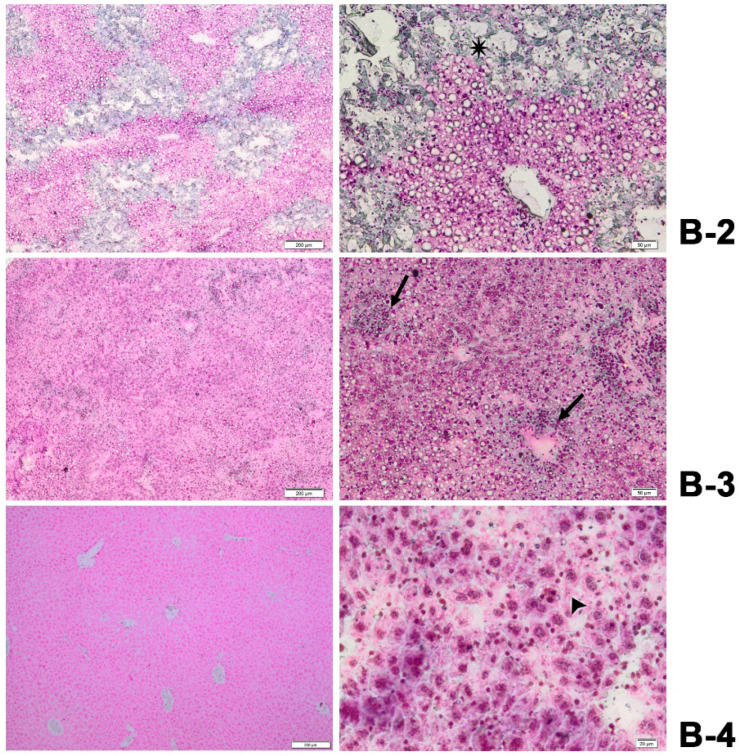
Effects of *Berberis vulgaris* extract/β-cyclodextrin on CCl_4_-induced DNA damage. (**A**) Lane 1-DNA isolated from normal liver; Lane 2: DNA isolated from CCl_4_ intoxicated liver; Lane 3: DNA isolated from liver treated with *Berberis vulgaris*; Lane 4: DNA isolated from liver pre-treated with *Berberis vulgaris* extract followed by i.p. CCl_4_ injection; Lane 5: DNA isolated from liver treated with *Berberis vulgaris* extract/β cyclodextrin; Lane 6: DNA isolated from liver pre-treated with *Berberis vulgaris* extract/β cyclodextrin followed by i.p. CCl_4_ injection; Lane 7: Marker (1-kb DNA ladder). (**B**) Methyl-green pyronin staining of liver. (**B-1**): Control group; (**B-2**): CCl_4_ group; extended ssDNA areas (asterix); (**B-3**): *Berberis vulgaris* extract + CCl_4_; semnificative reduction of ssDNA areas (arrow); (**B-4**): *Berberis vulgaris* extract/β cyclodextrin + CCl_4_; ssDNA areas loss and normal RNA distribution (arrowhead) (DNA—pale green; RNA—pink).
